# Evaluation of the In Vitro Cytotoxic Activity of Ursolic Acid PLGA Nanoparticles against Pancreatic Ductal Adenocarcinoma Cell Lines

**DOI:** 10.3390/ma14174917

**Published:** 2021-08-29

**Authors:** Adam Markowski, Paweł Migdał, Adrianna Zygmunt, Magdalena Zaremba-Czogalla, Jerzy Gubernator

**Affiliations:** 1 Department of Lipids and Liposomes, Faculty of Biotechnology, University of Wroclaw, Joliot-Curie 14a, 50-383 Wroclaw, Poland; adrianna.zygmunt@uwr.edu.pl (A.Z.); magdalena.zaremba-czogalla@uwr.edu.pl (M.Z.-C.); 2 Ludwik Hirszfeld Institute of Immunology and Experimental Therapy, Polish Academy of Science, Weigla 12, 53-114 Wrocław, Poland; pawel.migdal@hirszfeld.pl

**Keywords:** pancreatic cancer, nanoparticles, PLGA, nanocarriers, terpenoids, naturally derived compounds, ursolic acid

## Abstract

Among all the types of cancer, Pancreatic Ductal Adenocarcinoma remains one of the deadliest and hardest to fight and there is a critical unmet need for new drugs and therapies for its treatment. Naturally derived compounds, such as pentacyclic triterpenoids, have gathered attention because of their high cytotoxic potential towards pancreatic cancer cells, with a wide biological activity spectrum, with ursolic acid (UA) being one of the most interesting. However, due to its minimal water solubility, it is necessary to prepare a nanocarrier vehicle to aid in the delivery of this compound. Poly(lactic-*co*-glycolic acid) or PLGA polymeric nanocarriers are an essential tool for ursolic acid delivery and can overcome the lack in its biological activity observed after incorporating within liposomes. We prepared UA-PLGA nanoparticles with a PEG modification, to achieve a long circulation time, by using a nanoprecipitation method and subsequently performed an MTT cytotoxicity assay towards AsPC-1 and BxPC-3 cells, with TEM visualization of the nanoparticles and their cellular uptake. We established repeatable preparation procedures of the nanoparticles and achieved biologically active nanocarriers with an IC50 below 30 µM, with an appropriate size for intravenous dosage (around 140 nm), high sample homogeneity (below 0.2) and reasonable encapsulation efficiency (up to 50%). These results represent the first steps in the development of potentially effective PDAC therapies based on novel biologically active and promising triterpenoids.

## 1. Introduction

Despite all efforts from years of research and development, pancreatic cancer (PC) remains one of the deadliest groups of cancers with very low treatment efficiency and poor prognosis [1]. Based on the Globocan 2020 reports, it ranks seventh in the world and fourth in Europe among the leading causes of cancer-related deaths. The vast majority of PCs, nearly 90%, are Pancreatic Ductal Adenocarcinomas (PDAC), which is considered one of the deadliest cancers of the digestive system [2]. It is predicted that, by 2030, PDAC will be the third cancer-related cause of death in the USA [3]. There are a number of reasons responsible for this phenomenon. One of these is a very poor and mostly inaccurate diagnostic process, arising from the long asymptomatic progression of the disease in its early stages. The vast majority of PDAC diagnoses are made in the late or final stages of cancer progression, where the tumor is mostly unamenable to resection and, what is more important, increased PDAC metastases are already present at this stage, mostly predominantly located in the liver and lungs. The second reason responsible for PDAC mortality is that this type of cancer is highly resistant to therapy, due to its rich extracellular matrix component [4,5,6]. Currently, we only have limited options for PDAC treatment, with most of them based on chemotherapy based on cytostatics, such as gemcitabine or nab-paclitaxel, or the more complex drug system, FOLFIRINOX, a combination of folinic acid (FOL), 5-fluorouracil, (5-FU) irinotecan (IRIN) and oxaliplatin (OX). However, none of these therapies provides any satisfactory results in tumor regression, merely prolonging lifespan for a few months with many undesirable side effects, as a toll [7,8,9,10]. Based on these facts and state of knowledge, it is necessary to find new ways of treatment to overcome the high mortality of PDAC and most importantly, to discover effective drugs for this type of cancer.

One of the common strategies in cancer treatment is based on using nanocarriers for improved and targeted delivery of therapeutic agents. The best examples are liposomes, with the widely used and FDA-approved lipid-based nanocarriers, such as Doxil (liposomal doxorubicin) and Ambisome (liposomal amphoceritine B) as representative products [11]. There are numerous advantages of using nanocarriers in cancer therapy, such as the ability to deliver hydrophobic compounds and enhancing their bioavailability, pharmacokinetic proprieties, improving therapeutic effect of the drug via the accumulation of the nanocarriers within cancerous masses, due to the EPR (Enhanced Permeability and Retention) effect, and also lowering the side effects and toxicity of the drugs [12]. The other types of nanocarriers are those based on lactic and glycolic acid polymers. PLGA (poly-d,l-lactide-co-glycolide) nanoparticles are one of the most successfully used nanocarrier systems in the drug-delivery and biomaterials industry. Their key asset is very low toxicity, due to hydrolysis in the body to non-toxic monomers, H_2_O and CO_2_ [13]. It is also possible to modify the surface of PLGA nanoparticles with PEG, heparin, or specific targeting ligands, to enhance drug circulation in the bloodstream and their therapeutic effect [14]. Different methods of preparing PLGA nanoparticles can create an opportunity for encapsulation of various anticancer drugs, with confirmed encapsulations of paclitaxel, doxorubicin, cisplatin and 5-fluorouracil [13]. With continuing approval of the FDA for PLGA-based nanomedicines, these nanocarriers can be promising alternatives to liposomal drug delivery systems in situations where the encapsulation of certain compounds in liposomes is either inefficient or impossible.

Many medicinal plant-derived compounds were tested against pancreatic cell lines, with some of them reported to exhibit high cytotoxic potential against PC cells. Terpenoids are a subclass of natural products that are used in the treatment of skin, lung, colon and prostate cancer [15]. Some, such as docetaxel or paclitaxel, are used in chemotherapy, as apoptosis activators [16]. Other terpenoids are reported to display various anticancer-specific proprieties, such as the inhibition of Nf-kB signaling [17,18,19], stimulation of proapoptotic caspase-3 and 9 [20], targeting DNA damage [21] and stimulation of apoptosis in PC cells [22]. 

Ursolic Acid (UA) is a triterpenoid, containing six isoprene units, which occurs in a wide variety of medical plants, including rosemary, holy basil, blueberries, cranberries, olives, heather flower and other higher plants [23,24]. UA possesses a wide range of anti-cancer properties, for example, caspase activation [25,26], c-Jun N-terminal kinases (JNK) inhibition [27], downregulation of antiapoptotic genes [28,29], inhibition of COX-2 [30], and suppression of MMP-9 [31]. UA can also inhibit signal transduction and activation of transcription-3 (STAT-3) and Nf-kB, two key cancer-related cell signaling molecules, strictly correlated with PDAC development [32,33]. UA can also induce cell death via increasing the level of reactive oxygen species (ROS) [34] and, in some animal models, UA is found to be chemopreventive [35,36]. It has been confirmed that UA can inhibit PDAC cells via suppression of Nf-kB and STAT3 signaling and multiple inflammatory gene products connected with these two pathways. UA can also enhance the therapeutic effect of gemcitabine, which could be beneficial through using UA as a supporting therapy, or through a direct combination of UA with gemcitabine, as single chemotherapy [23].

In this study, we investigated three different PLGA-based nanoparticles with encapsulated UA. The first type of these nanoparticles are plain PLGA nanoparticles. The second type is made of PLGA along with a covalently attached PEG-2000 residue. The third type is nanoparticles containing an attached PEG-5000 residue. PEGylation in nanocarriers is necessary to prevent the rapid blood clearance of nanoparticles [37]. PEGylation can also enhance the accumulation of nanoparticles within the tumor mass, via the EPR (Enhanced Permeability and Retention) effect and aid in their penetration through the extracellular matrix. After preparation of these nanoparticles, we determined their size, polydispersity index and zeta potential. We also provided some TEM microscopy analysis and, most importantly, we investigated their cytotoxic effect towards two PDAC cell lines, namely, AsPC-1 and BxPC-3, to prove, that we prepared and obtained biologically active nanocarrier formulations that were active against their cellular targets in vitro, providing the basis for further evaluating these formulations for intravenous UA delivery for potentially effective PDAC treatment in vivo.

## 2. Materials and Methods

### 2.1. Chemicals and Reagents

PLGA Resomer^®^ RG 503 H, Poly(d,l-lactide-*co*-glycolide), 50:50, Mw 24,000–38,000 was acquired from Evonik, Essen, Germany. PLGA-PEG 2000 (PEG average Mn 2000, PLGA average Mn 11,500, lactide:glycolide 50:50) and PLGA-PEG 5000 (PEG average Mn 5000, PLGA Mn 20,000, lactide:glycolide 50:50) were purchased from Merck, Darmstadt, Germany. Ursolic acid was purchased from Wuxi Cima, China. Pluronic F-127 and Thiazolyl Blue Tetrazolium Bromide were purchased from Merck, Germany. RPMI-1640 cell culture media was purchased from Lonza, Basel, Belgium., Fetal bovine serum, GlutaMAX™ (L-alanyl-L-glutamine dipeptide in 0.85% NaCl) and 100× antibiotic-antimycotic were purchased from Life Technologies (Gibco/Life Technologies, Warsaw, Poland). Dimethylsulfoxide (DMSO) was purchased from ChemPur, Piekary Śląskie Poland. Uranyl acetate and copper mesh (400 Mesh) with formware filter and carbon shell, were purchased from Agar Scientific, Essex, UK.

### 2.2. Nanoparticles Preparation

Nanoparticles were prepared by a nanoprecipitation method. Polymers and UA were dissolved in DMSO and mixed together as an oil phase. Then, this oil phase was added dropwise into a 5% Pluronic F-127 solution, with stirring, at a temperature of 60 °C. After formation, the nanoparticles were cooled down to RT, and centrifuged twice, using a Sigma 3–30 KS centrifuge (25,000 RPM, RT) (Sigma, Osterode am Harz, Germany). After each centrifugation, pellets were washed and resuspended in MILIQ ultrapure water. After the final centrifugation, the nanoparticles were ready for further analysis.

### 2.3. Determination of Nanoparticles Size and Zeta Potential

Size, polydispersity (PDI) and zeta potential were measured using a Malvern NanoZS dynamic light scattering system (Malven Industries, Malvern, UK). Measurements were made in ultrapure MILIQ water under RT conditions. DLS measurement graphs are made by using built-in, averaging software, to acquire a single sample peak, made from three separate runs (*n* = 3).

### 2.4. Determination of UA Encapsulation Efficiency (EE)

Encapsulation efficiency was determined by measuring the UA concentration in the final nanoparticle suspensions, after two centrifugations and resuspension in the same volume of ultrapure MILIQ water as the initial sample volume. The UA concentration in the final PLGA suspensions was established using a Waters 600 HPLC system with a Phenomenex Kinetex C18 column (Phenomenex, Torrance, CA, USA)., (100 cm × 2 mm). Isocratic elution was performed over 10 min using an 80:20 acetonitrile:methanol composition at a flow rate of 1 mL/min. The HPLC system was equipped with a UV detector set to 210 nm.

### 2.5. Nanoparticle Stability Evaluation

The size, PDI and zeta potential of loaded and unloaded UA-nanoparticles were measured immediately after preparation (t = 0) and after storage at 4 °C for 30 days.

### 2.6. Analysis of UA-Nanoparticles by Transmission Electron Microscopy (TEM)

Visualisation of UA-PLGA nanoparticles was performed using a JEOL 1200 electron microscope (Jeol, Peabody, IN, USA). A total of 10 µL of nanoparticles suspended in ultrapure MILIQ water was applied on copper grid 400 mesh. After one minute, any excess of the sample was removed, and sample contrasting was performed in the presence of 2% uranyl acetate for one minute under a current of 80 kV.

### 2.7. Cell Culture

AsPC-1 (from ascites of a patient with PDAC) and BxPC-3 (primary pancreatic tumor) cells (ATCC. Manassas, VA, USA) were maintained with RPMI-1640 medium supplemented with 10% heat-inactivated fetal bovine serum (FBS), antibiotic-antimycotic mixture and GlutaMAX™ solution, under aseptic conditions in a Memmert ICO150 Med incubator (Memmert, Schwabach, Germany). Cultures were maintained at 37° C in a humidified atmosphere containing 5% CO_2_.

### 2.8. MTT Cell Viability Assay

The effect of UA-PLGA and PEGylated UA-PLGA nanoparticles was determined using a quantitative colorimetric MTT assay adapted from Mosmann [38]. Cells were seeded in 96-well plates (4500 cells per well), in an appropriate complete cell culture medium, for 24 h. Cells were treated with UA encapsulated in PLGA nanoparticles and UA dissolved in DMSO in the range of 2.5–80 µM (an equivalent volume of DMSO was used as a negative control, maximal concentration was 0.18% *v*/*v*), or control unloaded nanoparticles, for 72 h. The medium containing the tested formulations was removed and MTT solution (working solution: stock 0.5 mg/mL was 10 times diluted in medium) was added to the wells, and the plates were incubated for a further 3 h. Subsequently, the MTT solution was replaced with DMSO (50 µL/well) to dissolve the purple formazan crystals. Absorbance was measured at 560 nm, with a reference wavelength of 670 nm, on an Asys UVM 340 Microplate Reader (Cambridge, UK). Results were expressed as the percentage of surviving cells, with respect to the control (the untreated cells), calculated as:

Cell Viability (%) = (AT/AC) × 100,(1)

where:

AT = Absorbance of the treatment well (treated cells);

AC = Absorbance of the control well (untreated cells).

IC50 values were calculated using GraphPad Prism for Windows (GraphPad Software, La Jolla, CA, USA).

### 2.9. Cellular Uptake

Cellular uptake of Rhodamine 6G loaded PLGA-PEG 2000 nanoparticles by AsPC-1 and BxPC-3 cells were assessed by fluorescence microscopy. Rhodamine 6G was encapsulated into nanoparticles using exactly the same procedure as used for UA. Cancer cells were seeded onto glass cover slides placed in 24-well culture plates. After 24 h incubation, the cell culture medium was replaced with a medium containing Rhodamine 6G loaded PLGA nanoparticles. The cells were incubated at 37 °C for 2 h. Subsequently, cells were washed three times with PBS (37 °C), to remove excess nanoparticles, and fixed for 20 min in 4% paraformaldehyde, washed with Phosphate-buffer saline (PBS) and stained with DAPI. Slides were analyzed using a Leica TCS SP8 confocal microscope (Leica-Microsystems, Mannheim, Germany) with an HC PL APO CS2 63×/1.40 oil objective. To excite Rhodamine and 4′,6-diamidino-2-phenylindole (DAPI), a fluorescent probe which forms a complex by fixing to DNA, 561 nm and 405 nm lasers (Leica-Microsystems, Mannheim, Germany) were used, respectively.

### 2.10. Statistical Analysis

Data were presented as mean ± standard deviation. Statistical analyses were made using GraphPad Prism software (Version 7, Graphpad Software, San Diego, CA, USA). with a one-way ANOVA (Prism 7 for Windows) and Dunnett’s multiple comparisons test. A *p*-value equal to or less than ≤0.05 was considered statistically significant.

## 3. Results

### 3.1. UA Encapsulation and Morphology Parameters Evaluation

**Figure 1:**
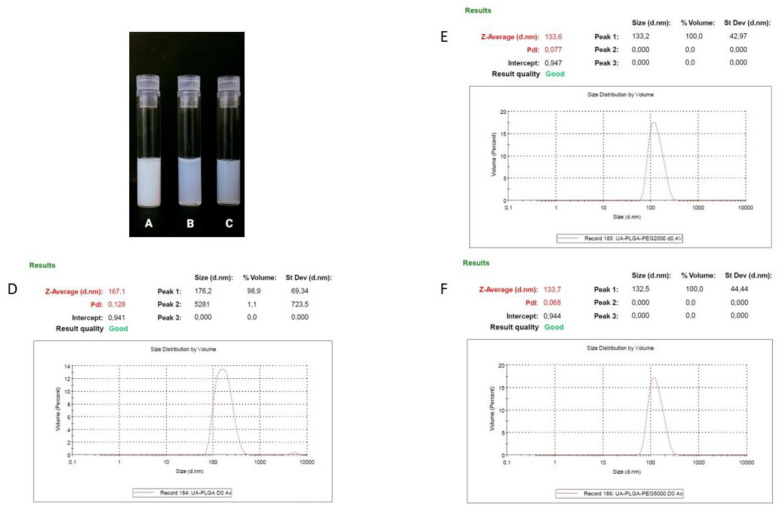
Visual appearance of the UA encapsulated nanoparticles and DLS averaged measurements results (*n* = 3) for each of the UA nanoparticles, with size [nm] and PDI values shown. (**A**,**D**). UA-PLGA, (**B**,**E**). UA-PLGA-PEG 2000, (**C**,**F**) UA-PLGA-PEG 5000.

Ursolic acid, due to its extreme hydrophobic nature (class IV of the Biopharmaceutics Classification System), is inappropriate in its non-formulated form for intravenous administration [39]. That is why we established a nanocarrier for the potential delivery of UA. A number of liposomal formulations of UA were prepared in our laboratory, but none of them exhibited any significant biological activity towards pancreatic cell lines (data not shown). That is why we established alternative nanocarrier formulations suitable for intravenous administration. Nanoparticles were prepared using a nanoprecipitation method, involving a simple one-step, manufacturing and saleable method. We prepared three different PLGA-based nanoparticles and evaluated them in terms of size, polydispersity index (PDI), zeta potential and encapsulation efficiency. As shown in Table 1, dynamic light scattering (DLS) results indicated that the diameter of the nanocarriers ranged between 133.7 ± 0.8 nm for UA-PLGA-PEG 5000 to 167.1 167.1 ± 1 nm for non-PEGylated UA-PLGA. Additionally, PDI values ranged from 0.052 to 0.128, with Zeta-potentials ranging from –30.4 ± 2.9 to −18.1 ± 1. Unloaded nanoparticles were also prepared and measured. The encapsulation efficiency (EE%) for UA loading into nanoparticles was also determined. EE% was similar for all three formulations with values ranging from 43.1% ± 5.3 for UA-PLGA-PEG 5000 to 47.4% ± 10.5 for UA-PLGA. The results of these analyses are presented in Table 1. Figure 1 presents the visual appearance of the nanoparticles with encapsulated UA and DLS measurement graphs.

### 3.2. TEM Visualization of Nanoparticles

**Figure 2:**
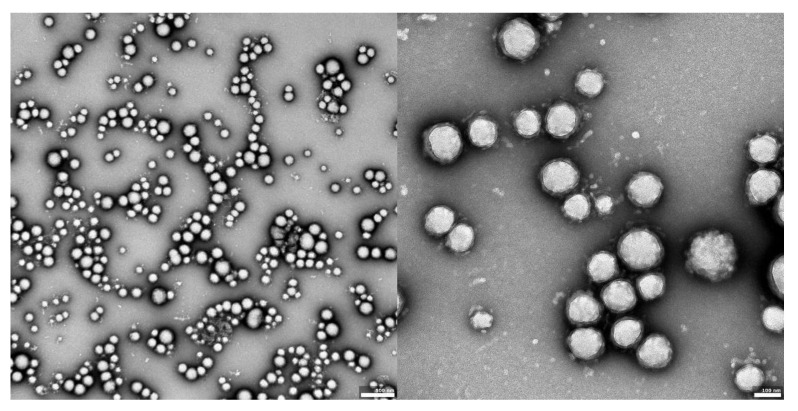
TEM images of UA-PLGA nanoparticles: **left panel**: UA-PLGA magnitude 10 k, **right panel**: UA-PLGA Magnification: 40 k. Scale bar 500 nm (**left**), 100 nm (**right**).

**Figure 3:**
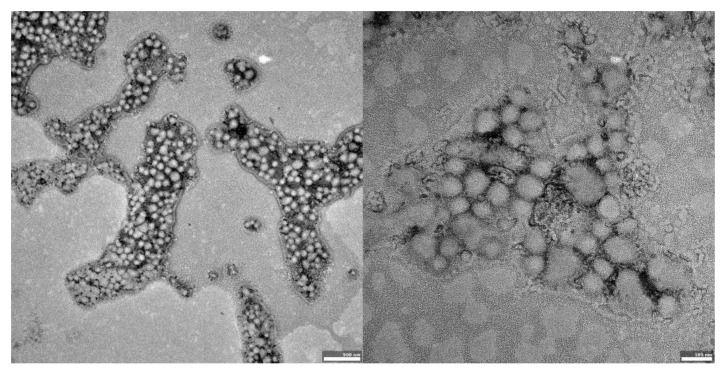
TEM images of UA-PLGA-PEG 2000 nanoparticles. **Left panel**: UA-PLGA-PEG 2000 magnitude 12 k, **right panel**: UA-PLGA-PEG 2000 Magnification: 50 k. Scale bar 500 nm (**left**), 100 nm (**right**).

**Figure 4:**
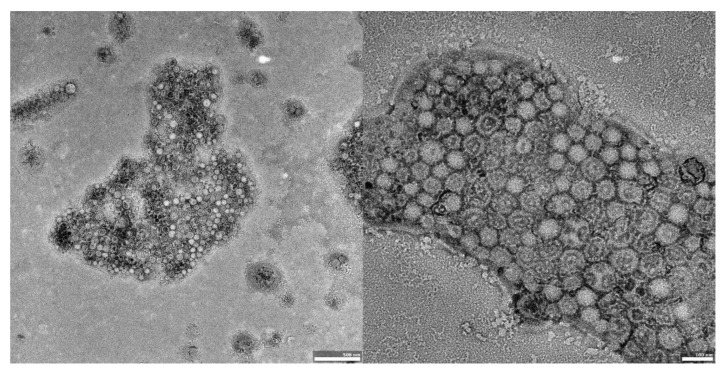
TEM images of UA-PLGA-PEG 5000 nanoparticles. **Left panel**: UA-PLGA-PEG 5000 magnitude 15 k. **Right panel**: UA-PLGA-PEG 5000 Magnification: 40 k. Scale bar 500 nm (**left**), 100 nm (**right**).

The visual appearance of the UA nanoparticles in solution was translucent, similar to very diluted milk, but still transparent. For microscopic visualization, transmission electron microscopy was used. TEM images (Figure 2, Figure 3 and Figure 4) showed spherical, porous entities, with good homogeneity. UA-PLGA-PEG 2000 (Figure 3) showed a more deviation from the ideal sphere, but the sample still maintained sphere-like shapes with no aberrations in size or homogeneity. Less contrast was observed in the PEGylated samples (Figure 3 and Figure 4), compared to the highly-contrasted PLGA sample (Figure 2).

### 3.3. Assessment of UA and UA-PLGA Nanoparticle Toxicity towards Human Pancreatic Cancer Cell Lines

**Figure 5:**
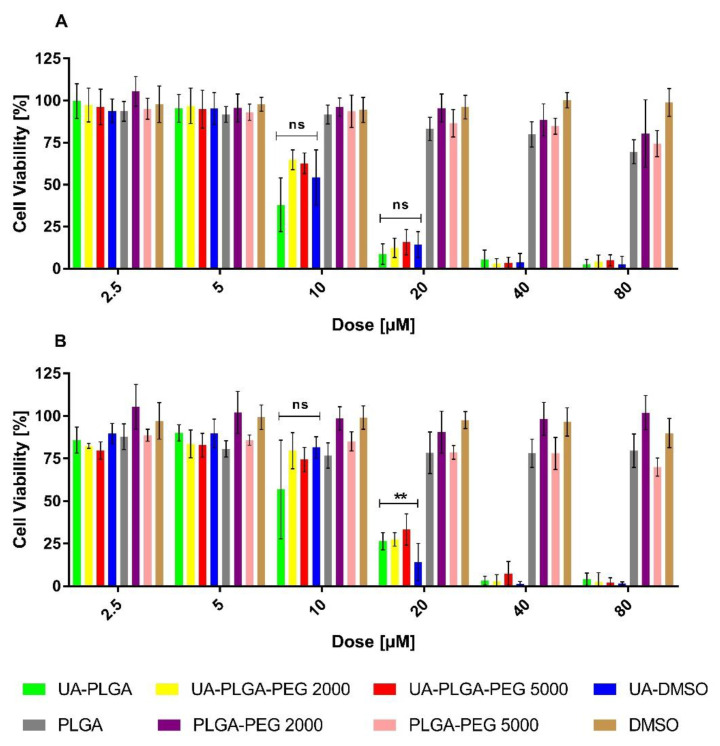
Cytotoxic effect of ursolic acid encapsulated in PLGA nanoparticles or in the free non-encapsulated form in DMSO, determined by the MTT assay, after 72 h of incubation, for AsPC-1 (**A**) and BxPC-3 (**B**) cell lines. For points 20 μM and 10 μM statistical significance between free and loaded compound was evaluated by Graphpad Prism 7 and was shown, as stars (**) represents significant difference, with *p*-value = 0.004. Ns stands for “non significant”.

To evaluate the anticancer potential of the UA and UA-PLGA nanoparticles, we investigated their in vitro cytotoxicity against two human pancreatic cancer cell lines (AsPC-1 and BxPC-3). During experiments, cells were incubated for 72 h with UA DMSO solution (free compound), DMSO (solvent control) or UA loaded into nanoparticles as well as unloaded nanoparticles (without UA). The experimental outcome was established using the MTT test, which is based on the detection of the oxidoreductive enzymes (especially succinate dehydrogenase) in the mitochondria of living, fully metabolizing cells. During the experiment, cells were incubated with a range of concentrations (2.5–80 µM) of UA dissolved in DMSO (which is commonly used as a solvent for drug testing), which was treated as a positive control, or UA encapsulated in PLGA nanoparticles. As negative controls, pure DMSO or “empty” nanoparticles were used. The results are presented in Figure 5.

The results showed a dose-dependent anticancer effect of UA either as a “free” compound or encapsulated in PLGA. What is worth to mention, UA-loaded nanoparticles exhibit similar anticancer activity as an unencapsulated compound. The IC50 value, which is a measure of biological activity, was very similar between every sample tested, ranging between 10.1 to 14.2 µM, and no major differences were observed between the two cell lines tested. Individual IC50 values for each sample against the two cell lines are shown in Table 2.

### 3.4. Preliminary Stability of UA Nanoparticles

It is important to establish the long-term stability of nanocarriers under storage, to determine any potential disruptions in the morphology of the samples. We measured the size, PDI and zeta potential of each sample immediately after preparation, and after 33 days of storage at 4 degrees. The nanoparticles increased in size after 33 days of storage. For UA-PLGA, the increase in size was 15 nm while, for both UA-PLGA-PEG 2000 and 5000, this difference was ~25 nm. Additionally, the zeta potential increased for UA-290 PLGA and UA-PLGA-PEG2000 (i.e., becoming more negative) after 33 days of storage, but there was no major change with time in the zeta potential of UA-PLGA-PEG5000. However, with no major changes in the PDI, the interpretation of the data would predict some “swelling” effect for the nanoparticles, with no loss in terms of homogeneity. There was no evidence of aggregation or any fusion events between the nanoparticles in the samples tested. Table 3 presents size, PDI and zeta values at the beginning of the measurements, and after storage for 33 days.

### 3.5. Cellular Uptake of UA-PLGA-PEG 2000 Nanoparticles

**Figure 6:**
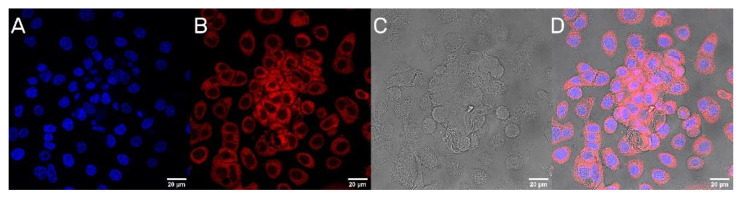
Visualization of the cellular uptake of Rhod6G loaded PLGA-PEG2000 nanoparticles by AsPC-1 pancreatic cell lines. (**A**). DAPI (**B**). Rhod6G fluorescence signal (**C**). transmitted light and (**D**). the merged image of the cells was observed after 2 h of incubation with labeled particles. Scale bar = 20 µm.

**Figure 7:**
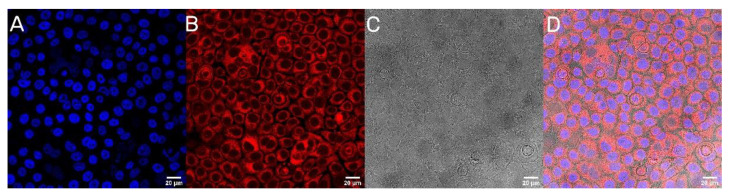
Visualization of the cellular uptake of Rhod6G loaded PLGA-PEG2000 nanoparticles by BxPC-3 pancreatic cell lines. (**A**). DAPI (**B**). Rhod6G fluorescence signal (**C**). transmitted light and (**D**). the merged image of the cells was observed after 2 h of incubation with labeled particles. Scale bar = 20 µm.

The next step was to evaluate the cellular uptake of the nanoparticles. For this purpose, we labeled nanoparticles with Rhodamine 6G, which is commonly used for bioimaging studies [37]. Confocal microscopy observation was performed using fluorescence signals from two fluorophores: one from cells nuclei stained with DAPI, the second from Rhodamine 6G encapsulated in nanoparticles, with the addition of transmitted light as well. After 2 h of incubation, the PLGA-PEG2000 nanoparticles were effectively internalized within AsPC-1 and BxPC-3 cells (Figure 6 and Figure 7).

## 4. Discussion

Despite all of the efforts made in PDAC therapy, this type of cancer still leads to one of the deadliest cancers, being highly chemoresistant, hard to diagnose and with high recurrence and metastatic potential [3]. Consequently, there is much work to do in term of developing new drugs and therapies against PDAC, especially to achieve complete remission or cure. One key strategy for such an aggressive disease is to use naturally-derived compounds that possess a wide spectrum of anticancer activities that help to overcome multidrug resistance [40,41], prevent metastasis [42], inhibit precancerous signaling pathways [43,44,45], or possess direct cytotoxic effects towards cancer cells [46,47]. Unfortunately, many of these types of naturally-derived compounds also show poor bioavailability, due to their highly hydrophobic nature which, in turn, disqualifies them for direct intravenous usage without specific formulation [48]. 

Ursolic acid, as a member of a large subclass of naturally-derived terpenoids, possesses many interesting anticancer properties, such as direct cytotoxic, antimetastatic, and chemopreventive effects [35,36,49]. As mentioned, UA can be highly effective in PDAC, especially in combination with gemcitabine, which can overcome the very poor response from gemcitabine therapy alone [23]. Additionally, with successful liposomal encapsulation of gemcitabine reported, it is possible to consider the application of combined therapy of UA and gemcitabine, based on nanoformulations [50], to enhance treatment efficiency even further.

PLGA-based nanoparticles are emerging as promising alternatives to liposomes as pharmacological carriers, because of the suitable characteristics they possess for intravenous administration, biocompatibility, the possibilities for surface modifications and because of their non-toxic properties since they are metabolized to carbon dioxide and water in humans [51,52,53,54]. Similar to liposomes, PLGA-nanoparticles offer sustained and targeted delivery, especially with PEG modifications. Further, the possibility for oral, pulmonary, or intravenous routes of administration provides many opportunities and approaches to achieve the optimal biological effect for the system [55,56].

As the method of nanoparticle preparation, we chose the nanoprecipitation method, with polymer and ursolic acid dissolved in DMSO as the oil phase, and 5% Pluronic F-127 as the aqueous phase. This one-step method is characterized by good reproducibility, scalability, with controllable preparation conditions, but with a relatively low encapsulation efficiency [57]. However, this method can provide nanoparticles with good morphological characteristics, such as size and polydispersity, that are suitable for potential intravenous usage [58]. Our procedure was successful in obtaining three types of ursolic acid-loaded nanoparticles: plain PLGA nanoparticles and two types of PEGylated nanoparticles, with corresponding, unloaded particles. Every fresh UA-based formulation represented good values of size and excellent homogeneity, ranging between 132 to 168 nm, with PDI values below 0.2. An encapsulation efficiency between 43.1% to 47.5% was achieved for ursolic acid with the reported preparation method. According to the literature, a very similar UA nanoparticle preparation procedure was presented by Merlin et al., where the researchers obtained nanoparticles with similar size and PDI values, but higher encapsulation efficiency, probably due to the use of a different method of determining UA concentration, with HPLC being recognized as a more accurate method for measuring non-chromophore rich compounds [59]. According to the literature, PLGA based nanoparticles are characterized by negative values of zeta potential, which are considered suitable for intravenous dosage. However, even without interaction between carrier and serum proteins, negatively charged carriers can still induce immunological reactions. To prevent this phenomenon, PEG is widely used in the liposomal or polymeric carriers industry. Our UA-PLGA nanocarriers are characterized both by negative zeta potential values and by PEG 2000 or 5000 addition. The addition of PEG residue did not change the negative charge of the carrier, but according to the literature, it will prevent interaction with the immune system, similar to STEALTH liposomes [60]. Other ursolic acid encapsulation procedures describe single- or multi-emulsion solvent evaporation methods. The authors achieved similar values of encapsulation efficiency and size, but higher PDI values, especially for multi emulsion solvent evaporation [61]. Another trial describes the encapsulation of UA and oleanolic acid (OA) with a mixture of plant-derived extracts containing natural terpenoids for the treatment of ocular inflammatory events. These nanoparticles were characterized by good encapsulation efficiency (almost 80%) but with a lower ratio PLGA/compound and higher particle size, making them unsuitable for intravenous delivery. However, it is worth mentioning that the particles prepared by Alvarado waste al were never intended for intravenous usage [62].

Further, in combination with the low IC50 values of UA (between 10.10 and 14.2 µM), with limited toxicity coming from the nanocarrier itself, these encapsulation efficiency values appear to be adequate for future potential therapy procedures based on this type of nanocarrier.

We propose our variation of preparation PLGA nanoparticles, based on available knowledge and protocols [57,63,64]. Using ultra-pure MILIQ water as the aqueous phase is associated with very unpredictable particle preparation, especially for plain PLGA nanoparticles. Exchanging water with 5% Pluronic F-127 results in a more repeatable procedure for preparing such nanoparticles. Pluronic F-127 acts as a surfactant. It negates any interactions between nanoparticles during formation, especially non-PEGylated PLGA nanoparticles. It results in higher homogeneity of the samples. Other additional steps, such as heating and mixing with high speed (1500 RPM), also helped in establishing more stable and reproducible sample preparations.

TEM photographs show populations of homogenous, spherical-shaped nanoparticles, as was predicted, with a similar visual appearance to the nanoparticles described by Baisha et al. [60]. The UA-PLGA-PEG 2000 formulation showed a little more variability in a sphere shape, being more ellipsoidal or, “egg-shaped”. The lower contrast in the PEGylated samples could be correlated with a slightly lower contrasting efficiency with 2% uranyl acetate, but this requires further investigation. Significantly, no unwanted phenomena were observed, such as breakage, collapse, or structural disturbances in any form of the samples. We also did not observe any UA precipitation, which can be seen as crystal-like entities in microscopy images. To this date, we do not know of any other research group that has prepared PEGylated ursolic acid nanoparticles. Saneja et al. prepared PEGylated nanoparticles containing another triterpenoid, betulinic acid, towards the PANC-1 pancreatic cancer cell line, but with a synthesis totally prepared by them. These nanoparticles were not prepared using commercially available polymers [65].

Another critical parameter of nanocarriers is the stability of the obtained vesicles. This is especially important, considering future pharmaceutical or industrial development of this technology because any nanocarrier formulation should display long-term stability without any trace of aggregation, loss of structure, or drug precipitation [66]. We did not observe any indications of sample disruption or vesicle damage during the 33 days of stability testing conducted as part of this study. In general, formulation maintains homogeneity and integrity, despite changes in size and zeta potential values. Moreover, we did not observe any signs of aggregation or separation in the samples.

A final point of our work was to evaluate the cytotoxic potential of our nanocarriers. As we mentioned before, our first attempt was to prepare liposomal formulations of ursolic acid. However, none of our liposomal UA samples were active towards pancreatic cancer cells. To this date, we could not answer this phenomenon. One of our hypotheses is very strong interactions between UA and phospholipids, which negates the cytotoxic potential of UA. However, to this date, there are few published liposomal formulations of UA, where the triterpenoid did not lose its cytotoxicity towards cells [67,68,69,70]. Yet, there is no liposomal formulation of UA used in potential PDAC treatment, maybe because of this unknown phenomenon. This is the reason why we choose a different approach for delivering UA in nanoformulation. Our PLGA nanoparticles maintain the cytotoxic potential of UA, with IC50, ranged between 10.1 to 14.2 µM, which is lower than those reported in the literature for PDAC cell lines [71]. It is worth mentioning that the cytotoxicity comes directly from encapsulated UA via endocytosis of nanoparticles into cells, and not from accelerated hydrolysis of the particles in the cell medium. This event was confirmed by confocal microscopy, where nanoparticles were stained with Rhod6G. One of the main goals for future experiments will be to evaluate the specificity index of obtained nanoparticles. This would be necessary for the establishment of cytotoxicity levels towards non-cancerous cell lines like human skin fibroblasts (NHDF) of ursolic acid nanoparticles.

Lastly, we performed a statistical analysis of the data using Dunnett’s multiple comparisons test. We established two parameters for statistical consideration. The first parameter was establishing the statistical significance of empty nanocarriers toxicity. With a *p*-value less than, or equal to 0.05, our results are statistically relevant. However, we strongly believe, that this effect is correlated with the acidic character of the nanocarrier itself [72]. The second parameter was to investigate the difference between the toxicity of non-encapsulated UA and UA encapsulated within nanoparticles. For this, two concentrations, namely 20 and 10 µM, were evaluated in both cell lines. For the AsPC-1 line, no statistically significant difference was observed in toxicity between the non-encapsulated UA and UA-nanoparticle formulation at both concentrations so, in this case, we can assume the same cytotoxic potential of UA-PLGA nanoparticles as the ”free” drug, which is promising. For the BxPC-3 cell line, we did not observe any statistically significant difference in toxicity for the 10 µM dosage. However, we observed a statistically significant difference between non-encapsulated UA and UA-loaded nanoparticles at the 20 µM dosage. Considering this fact alongside the other factors, such as an overall similar IC50 value and cytotoxic potential, we consider this as being of low concern.

## 5. Conclusions

We prepared and evaluated three types of ursolic acid PLGA nanoparticles of the appropriate size and with excellent homogeneity, suited for intravenous usage. This is also the first instance of encapsulating UA into PEGylated nanoparticles, with two different PEG residue sizes, facilitating potential enhanced nanocarrier bloodstream circulation time and tumor targeting. High biological activity associated with good cellular uptake and in vitro evidence of the anticancer properties of ursolic acid with two pancreatic tumor cell lines, classify these nanoparticles as candidates for potential in vivo experiments, to establish their true potential as a novel and effective treatment of one of the most resistant human cancers today.

## Figures and Tables

**Table 1:** Nanoparticle characterization.

Sample	UA-PLGA	PLGA	UA-PLGA-PEG 2000	PLGA-PEG 2000	UA-PLGA-PEG 5000	PLGA-PEG 5000
Size	167.1 ± 1	171.9 ± 2.7	133.6 ± 0.7	142.6 ± 0.9	133.7 ± 0.8	132.1 ± 1.2
PDI	0.128 ± 0.01	0.052 ± 0.01	0.077 ± 0.02	0.096 ± 0.02	0.068 ± 0.02	0.066 ± 0.02
Zeta	−20 ± 0.8	−29 ± 0.2	−22.6 ± 2.8	−30.4 ± 2.9	−18.1 ± 1	−30.2 ± 5.4
Encapsulation efficiency [%]	47.4 ± 10.5	-	45.1 ± 6.5	-	43.1 ± 5.3	-

**Table 2:** IC50 values for encapsulated and non-encapsulated ursolic acid on two PDAC cell lines, AsPC-1 and BxPC-3.

Sample	AsPC-1 IC50 Value [µM]	BxPC-3 IC50 Value [µM]
UA-PLGA	10.1 ± 1	12.6 ± 4.5
UA-PLGA-PEG 2000	11.7 ± 0.6	14.1 ± 2.2
UA-PLGA-PEG 5000	11.9 ± 1	14.2 ± 2.7
UA-DMSO	11.1 ± 2.4	13.5 ± 1

**Table 3:** Preliminary stability results for the tested nanoformulations.

**Sample at Day 0**	**UA-PLGA**	**UA-PLGA-PEG 2000**	**UA-PLGA-PEG 5000**
Size [nm]	167.1 ± 1	133.6 ± 0.7	133.7 ± 0.8
PDI	0.128 ± 0.01	0.077 ± 0.02	0.068 ± 0.025
Zeta [mV]	−20 ± 0.8	−22.6 ± 2.8	−18,1 ± 0.9
**Sample at Day 33**	**UA-PLGA**	**UA-PLGA-PEG 2000**	**UA-PLGA-PEG 5000**
Size [nm]	182.1 ± 1.8	158.7 ± 1.6	158.4 ± 0.7
PDI	0.12 ± 0.02	0.097 ± 0.02	0.102 ± 0.2
Zeta [mV]	−27.2 ± 0.5	−26.4 ± 1	−18.4 ± 9.2

## Data Availability

The data are stored by corresponding author, and available by request.
